# Longitudinal Evaluation of Fatty Acid Metabolism in Normal and Spontaneously Hypertensive Rat Hearts with Dynamic MicroSPECT Imaging

**DOI:** 10.1155/2011/893129

**Published:** 2010-12-08

**Authors:** Bryan W. Reutter, Ronald H. Huesman, Kathleen M. Brennan, Rostyslav Boutchko, Stephen M. Hanrahan, Grant T. Gullberg

**Affiliations:** Department of Radiotracer Development & Imaging Technology, Lawrence Berkeley National Laboratory, One Cyclotron Road, Berkeley, CA 94720, USA

## Abstract

The goal of this project is to develop radionuclide molecular imaging technologies using a clinical pinhole SPECT/CT scanner to quantify changes in cardiac metabolism using the spontaneously hypertensive rat (SHR) as a model of hypertensive-related pathophysiology. This paper quantitatively compares fatty acid metabolism in hearts of SHR and Wistar-Kyoto normal rats as a function of age and thereby tracks physiological changes associated with the onset and progression of heart failure in the SHR model. The fatty acid analog, ^123^I-labeled BMIPP, was used in longitudinal metabolic pinhole SPECT imaging studies performed every seven months for 21 months. The uniqueness of this project is the development of techniques for estimating the blood input function from projection data acquired by a slowly rotating camera that is imaging fast circulation and the quantification of the kinetics of ^123^I-BMIPP by fitting compartmental models to the blood and tissue time-activity curves.

## 1. Introduction

**Figure 1:**
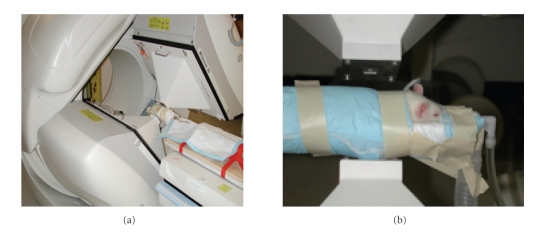
Clinical dual-detector SPECT/CT scanner with custom pinhole collimators used for quantitative dynamic imaging of fatty acid metabolism in the rat heart.

Hypertrophic cardiomyopathy is a condition in which the heart muscle becomes thick, forcing the heart to work harder to pump blood. Under normal conditions the heart uses glucose (~30%), fatty acids (~60%), and lactate (~10%) as primary energy sources, in addition to amino acids and ketone bodies [1–3]. In the case of cardiac hypertrophy, however, there is an increase in cardiac mass and a switch to a reliance on glucose metabolism. To be able to detect and interpret the early onset of this change, there is the need to develop methodology for sensitive predictors for early detection, prognosis and to follow the response to therapy for hypertrophic cardiomyopathy. In clinical settings, the abnormalities of fatty acid metabolism in hypertrophic cardiomyopathy can be recognized by the decreased uptake in single-photon emission computed tomography (SPECT) images [4]. It has also been demonstrated that compartmental analysis and dynamic SPECT imaging make it possible to detect abnormalities of fatty acid utilization earlier than SPECT imaging, with the potential to provide an even earlier prediction of the onset of cardiac hypertrophy [5]. There is a need to develop technology for imaging small animal models on clinical SPECT systems that could easily be translated to the clinic for diagnosis and management of patients with cardiac hypertrophy. However, the challenge is to perform compartmental analysis of dynamic studies in small animals with pinhole SPECT using slowly rotating gantries (slow camera rotation with 1 s per view) when the recirculation time in the animals is 6–8 s. This study was designed to follow the changes in fatty acid metabolism in the left ventricular myocardium associated with the progression of hypertrophy in the spontaneously hypertensive rat (SHR) and, in so doing, develop methodology for data acquisition and data processing techniques of pinhole SPECT acquired data [6–11]. The goal of this project is to develop radionuclide molecular imaging technologies using a clinical dual-modality pinhole SPECT/X-ray computed tomography (CT) scanner to quantify the changes in metabolism in the heart using the SHR as a model of hypertensive-related pathophysiology (Figure 1).

 The SHR (Okamoto and Aoki strain) has hypertension associated with generalized dyslipidemia and insulin resistance. It has been noted that the SHR model has a defective gene (CD36) on chromosome 4 [12]. The gene in the SHR results in a defective fatty acid enzyme (translocase), which functions in long-chain fatty acid transport into the cell. This compromises tissue utilization of fatty acid and increases the basal glucose metabolism and hyperinsulinemia.

Mechanisms involved in the progression of heart failure are believed to be, in part, related to alteration in myocardial energy metabolism [13–15]. Plasma levels of glucose, fatty acids, and lactate determine which of these substrates are oxidized [16]. In the failing heart, energy substrate utilization changes from fatty acid oxidation to one of glycolysis utilization [17, 18]. In the case of pressure overload, there is an increased reliance on carbohydrate oxidation in an attempt to maintain contractile function. The myocardial extraction and retention of fatty acids are impaired in the advanced stage of heart failure [19]. The fatty acid analog, **β**-methyl-p-[^123^I]-iodophenyl-pentadecanoic acid (^123^I-BMIPP), currently being evaluated in human studies to study the progression of heart failure, has been used to image the metabolism of fatty acids [1, 20]. Imaging with ^123^I-BMIPP in a canine has shown that myocardial fatty acid oxidation begins to be inhibited and that washout of ^123^I-BMIPP increases in the compensated stage of left ventricular dysfunction. Human, canine, and rodent studies show that in late-stage heart failure there is downregulation of myocardial fatty acid oxidation and accelerated glucose oxidation [21–23]. The reduction in fatty acid oxidation is not caused by changes in fatty acid availability in the blood [24]. The time course and the molecular mechanisms for this switch in substrate oxidation are not well understood [25, 26].

This paper quantitatively compares fatty acid metabolism in the hearts of SHR and Wistar-Kyoto (WKY) normal rats as a function of age, and thereby tracks physiological changes associated with the onset and progression of heart failure in the SHR model. ^123^I-BMIPP was used in longitudinal metabolic pinhole SPECT imaging studies performed every seven months for 21 months. The uniqueness of this project is the development of techniques for estimating the blood input function from projection data acquired by a slowly-rotating camera imaging fast circulation in a rat, and the quantification of the kinetics of ^123^I-BMIPP by fitting compartmental models to the blood input function and tissue uptake/washout time-activity curves (TACs).

In previous work, we addressed issues associated with reconstructing dynamic data acquired with use of a slowly-rotating camera [27–29]. The work presented here also addresses quantitative effects of limited spatial resolution in dynamic pinhole SPECT that result in underestimation of the metabolic rate of ^123^I-BMIPP in the rat myocardium. In particular, the partial volume effect blurs activity between the left ventricular blood pool and surrounding myocardial tissue and decreases contrast between the blood input and tissue uptake TACs [11, 30, 31]. Standard compartmental modeling straightforwardly accounts for the spillover of blood activity into tissue volumes [32]. However, accounting for the spillover of tissue activity into blood volumes is more problematic, and is a focus of the work presented here. Results are presented for imaging studies performed on two SHRs and two WKY normal rats at three ages.

This paper is organized as follows.  Section 2 begins by describing the data acquisition protocol for acquiring dynamic ^123^I-BMIPP data in a rat with use of a slowly-rotating dual-detector pinhole SPECT system. This section then provides overviews of our multiresolution methods for fully 3D reconstruction of a late static SPECT image (to determine the spatial locations for the left ventricular blood pool and myocardial tissue) and for fully 4-D reconstruction of an early dynamic SPECT image represented by 4-D splines that are piecewise constant in space and piecewise quadratic in time.  Section 2 concludes with details of our fully 4-D penalized least-squares reconstruction algorithm that uses a smooth 4-D image prior, as well as our methods for jointly estimating the blood input function and fatty acid metabolism from the reconstructed dynamic SPECT image.  Section 3 presents results obtained with use of these algorithms applied to dynamic SPECT imaging studies performed on two SHR and two WKY normal rats when the rats were age 7, 14, and 21 months (one SHR died before 21 months). The paper concludes with a discussion of the results and future work in Section 4.

## 2. Materials and Methods

### 2.1. Pinhole SPECT System Modeling and Data Acquisition

With use of methods described in [8, 9], dynamic cardiac pinhole SPECT projection data and pinhole geometric calibration data were acquired with slow gantry rotation on a dual-detector GE Millennium VG Hawkeye SPECT/CT scanner equipped with custom tungsten pinhole collimators having a 1.5 mm by 2 mm retangular aperture (Figure 1). The NaI(Tl) crystal in each detector has an area of 540 mm by 400 mm and is 9.5 mm thick. At an energy of 140 keV, the intrinsic spatial resolution is 3.9 mm and the energy resolution is 9.8%.

In the geometric configuration used for imaging rats (Figure 1), the pinhole collimators magnify the center of the field of view by a factor of about 4.8 on the faces of the large detectors. In a phantom study that we performed in this configuration [8, 9], the system could easily resolve the smallest features of the micro-Jaszczak phantom, which are 1.2 mm “cold” rod sources separated by 1.2 mm in a radioactive background [33]. Collimator response was modeled via ray tracing and excluded the effects of collimator penetration. The system model also excluded the effects of attenuation and scatter; however, we are currently studying these effects via Monte Carlo simulation in a separate investigation [34, 35].

**Figure 2:**
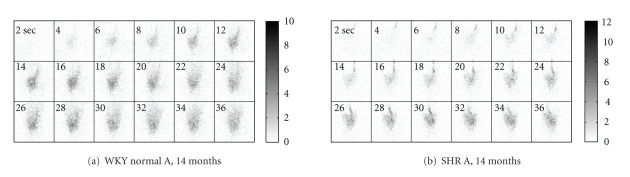
Dynamic cardiac ^123^I-BMIPP pinhole SPECT projection data acquired by one detector head during the interval 2–36 s for (a) a WKY normal rat and (b) an SHR. These early time frames show the arrival of the injected bolus at the heart, followed by initial uptake in the myocardium. The maximum numbers of counts in a detector bin are 10 and 12 for the WKY normal rat and SHR, respectively.

^123^I-BMIPP was obtained from Molecular Insight Pharmaceuticals. All imaging studies were performed in accordance with an Institutional Animal Care and Use Committee (IACUC) approved protocol. Rats were anesthetized throughout the entire procedure with use of 2–2.5% isoflurane inhalation anesthesia. A slow (10–30 s) injection of about 4 mCi (150 MBq) of ^123^I-BMIPP, via an IV catheter placed in the tail vein, was performed shortly after the dynamic data acquisition began. Data were acquired for 60 min in 1-s time frames with an angular step of 4 degrees per frame (Figure 2). For the biodistributions associated with these studies, overall system sensitivity was about 2000 cps/mCi (55 cps/MBq). The energy of the primary photopeak for ^123^I is 159 keV.

### 2.2. Multiresolution Fully 3D Late Static SPECT Image Reconstruction

To determine spatial locations for the left ventricular blood pool and myocardial tissue, late data acquired 1.5–60 min after injection were summed and a static image was reconstructed with use of a 3D version of the penalized least-squares image reconstruction that we describe in Section 2.4. The late static spatial distribution of ^123^I-BMIPP was modeled with use of 3D multiresolution spatial B-splines that were piecewise constant. The 3D spatial splines were organized on a 20 × 20 × 20 3D grid that provided uniform sampling of 3.2 mm in each dimension. Inside the volume containing the heart, a 6 × 6 × 6 neighborhood of these lower-resolution splines was replaced by a 12 × 12 × 12 neighborhood of higher-resolution splines that provided uniform sampling of 1.6 mm.

### 2.3. Multiresolution Fully 4-D Early Dynamic SPECT Image Reconstruction

**Figure 3:**
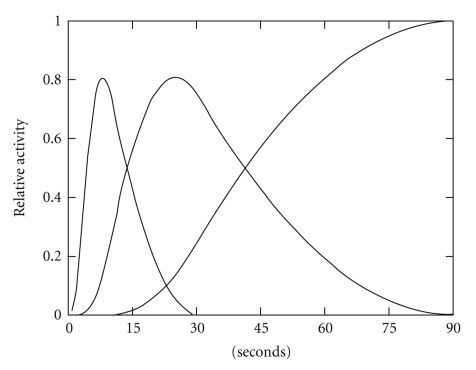
Piecewise quadratic temporal B-spline basis functions used to reconstruct dynamic data acquired during the first gantry rotation.

The time-varying spatial distribution of ^123^I-BMIPP was modeled with use of 4-D multiresolution B-splines that were piecewise constant in space and piecewise quadratic in time. The 4-D splines were spatially organized on a 10 × 10 × 10 3D grid that provided uniform sampling of 6.4 mm in each dimension. Inside the volume containing the heart, a 3 × 3 × 3 neighborhood of these lower-resolution splines was replaced by a 12 × 12 × 12 neighborhood of higher-resolution splines that provided uniform sampling of 1.6 mm. The 4-D splines were temporally organized on a 1D grid that provided nonuniform sampling intervals of 0–2.4, 2.4–9.4, 9.4–30, and 30–90 s during the first gantry rotation (Figure 3).

With use of the fully 4-D algorithm for penalized least-squares image reconstruction that we describe in Section 2.4, B-spline TACs for the multiresolution voxels were estimated directly from the dynamic pinhole SPECT projection data. This yielded estimates of temporal B-spline coefficients $\left\{{\hat{a}}_{mn};\;m=1,\ldots ,M;\;n=1,\ldots ,N\right\}$, where *M* is the number of spatial voxels and *N* is the number of temporal B-spline basis functions. The estimated TAC for the *m*th voxel is

(1) $${\hat{A}}^{m}\left(t\right)=\overset{N}{\underset{n=1}{\sum }}{\hat{a}}_{mn}V^{n}\left(t\right),$$

where *V* ^*n*^(*t*) are temporal B-spline basis functions (Figure 3).

### 2.4. Fully 4-D Penalized Least-Squares Reconstruction Algorithm with a Smooth 4-D Image Prior

A dynamic SPECT projection data model that relates detected events to a 4-D spatiotemporal B-spline representation of a time-varying radiotracer distribution can be written as

(2) p=Fa,

where **p** is an *I*-element column vector of modeled dynamic projection data values, **F** is an *I* × (*MN*) system matrix, **a** is an (*MN*)-element column vector of B-spline coefficients, and *I* is the total number of projection measurements acquired by the SPECT detectors. The system matrix **F** incorporates the spline model for time variation of the radiotracer distribution, as well as physical effects such as collimator response that affect detection of gamma rays emitted by the radiotracer distribution.

At the outset, the least-squares criterion to be minimized, *χ* ^2^, is simply the sum of squared differences between the measured projections, p*, and the modeled projections

(3) $$\chi^{2}={\left(p^{*}-Fa\right)}^{\text{T}}\left(p^{*}-Fa\right),$$

where the superscript “T” denotes the matrix transpose. Minimizing the criterion *χ* ^2^ yields an estimate, $\hat{a}$, of coefficients for the 4-D B-spline basis functions that represent the time-varying radiotracer distribution

(4) $$\hat{a}={\left(F^{\text{T}}F\right)}^{-1}F^{\text{T}}p^{*}.$$

The corresponding minimum value for the criterion *χ* ^2^ is

(5) $$\chi_{\min\;}^{2}={\left(p^{*}-F\hat{a}\right)}^{\text{T}}\left(p^{*}-F\hat{a}\right).$$

To reduce noise, we now wish to add a penalty term to the criterion *χ* ^2^ that encourages the reconstructed image to be smooth in both space and time. Insight into what a reasonable penalty term might be can be obtained by expressing *χ* ^2^ in terms of its minimum value

(6) $$\begin{array}{cc} \chi^{2} & ={\left(p^{*}-Fa\right)}^{\text{T}}\left(p^{*}-Fa\right)\\ & ={\left[\left(p^{*}-F\hat{a}\right)-F\left(a-\hat{a}\right)\right]}^{\text{T}}\left[\left(p^{*}-F\hat{a}\right)-F\left(a-\hat{a}\right)\right]\\ & =\chi_{\min\;}^{2}-2{\left(p^{*}-F\hat{a}\right)}^{\text{T}}F\left(a-\hat{a}\right)+{\left(a-\hat{a}\right)}^{\text{T}}F^{\text{T}}F\left(a-\hat{a}\right)\\ & =\chi_{\min\;}^{2}+{\left(a-\hat{a}\right)}^{\text{T}}F^{\text{T}}F\left(a-\hat{a}\right).\; \end{array}$$

Note that the term that is linear with respect to $\left(a-\hat{a}\right)$ vanishes because the model error $p^{*}-F\hat{a}$ lies in the null space of the backprojection operator **F** ^T^. Inspecting (6), one sees that differences from the least-squares solution $\hat{a}$ are penalized by the term ${\left(a-\hat{a}\right)}^{\text{T}}F^{\text{T}}F(a-\hat{a})$.

To mimic this effect for purposes of reducing noise, we propose to add a penalty term that resembles (**a** − **α**)^T^ **F** ^T^ **F**(**a** − **α**), where **α** is a smooth 4-D image prior obtained by normalizing a simple backprojection of the measured projections

(7) $$\alpha =\left(F^{\text{T}}p^{*}\right)\cdot /\left(F^{\text{T}}F\left[1\right]\right),$$

where the operator “·/” denotes pointwise division of elements in the left operand by the corresponding elements in the right operand and “[1]” denotes an (*MN*)-element column vector of ones. Note that normalization by **F** ^T^ **F**[1] ensures that backprojecting the noiseless projections of a constant image yields the original constant image. Note also that the image prior **α** has the desirable physiologic property of being nonnegative—thus, the reconstructed image is encouraged to have nonnegative 4-D B-spline coefficients.

The penalty term that we propose to use is

(8) $$\overset{I}{\underset{i=1}{\sum }}\;\overset{M}{\underset{m=1}{\sum }}\;\overset{N}{\underset{n=1}{\sum }}{\left[F_{i}^{mn}\left(a_{mn}-\alpha_{mn}\right)\right]}^{2},$$

where *F*_*i*_^*mn*^ is the [*i*, *m* + (*n* − 1)*M*]th element of the system matrix **F**, *a*_*mn*_ is the [*m* + (*n* − 1)*M*]th element of the spline coefficient vector **a**, and *α*_*mn*_ is the [*m* + (*n* − 1)*M*]th element of the smooth image prior **α**. For the resulting negatively correlated, zero-mean elements in the vector (**a** − **α**), (8) imposes a penalty that is greater than the penalty imposed by (**a** − **α**)^T^ **F** ^T^ **F**(**a** − **α**), and is more effective at suppressing “checkerboard” noise patterns. Note that the latter penalty can be expressed in summation notation as

(9) $$\overset{I}{\underset{i=1}{\sum }}{\left[\overset{M}{\underset{m=1}{\sum }}\;\overset{N}{\underset{n=1}{\sum }}F_{i}^{mn}\left(a_{mn}-\alpha_{mn}\right)\right]}^{2}.$$

In general, the penalties (8) or (9) may be scaled by a smoothing parameter **β**. The penalized least-squares criterion that we minimized for the work presented here is

(10) $$\begin{array}{cc} \Psi^{2} & =\overset{I}{\underset{i=1}{\sum }}{\left[p_{i}^{*}-\overset{M}{\underset{m=1}{\sum }}\;\overset{N}{\underset{n=1}{\sum }}F_{i}^{mn}a_{mn}\right]}^{2}\\ & \;+\beta \overset{I}{\underset{i=1}{\sum }}\;\overset{M}{\underset{m=1}{\sum }}\;\overset{N}{\underset{n=1}{\sum }}{\left[F_{i}^{mn}\left(a_{mn}-\alpha_{mn}\right)\right]}^{2}, \end{array}$$

where the first term on the right-hand side is the least-squares criterion (3) expressed in summation notation and the second term is the scaled penalty (8). The criterion Ψ^2^ is minimized by the following estimate, $\hat{a}$, of coefficients for the 4-D B-spline basis functions that represent the time-varying radiotracer distribution

(11) $$\hat{a}={\left[\left(1+\beta I\right)\cdot F^{\text{T}}F\right]}^{-1}\left[F^{\text{T}}p^{*}+\beta \mathrm{diag}\;\left(F^{\text{T}}F\right)\cdot \alpha \right],$$

where “1” is an (*MN*)×(*MN*) matrix of ones, **I** is an (*MN*)×(*MN*) identity matrix, “·” denotes the Hadamard product (i.e., pointwise multiplication of elements in the left operand by the corresponding elements in the right operand), and diag (**F** ^T^ **F**) is an (*MN*)-element column vector whose [*m* + (*n* − 1)*M*]th element is the [*m* + (*n* − 1)*M*]th diagonal element of **F** ^T^ **F**.

 Note that when the smoothing parameter **β** is zero, (11) simplifies to (4); whereas, the image estimate $\hat{a}$ approaches the smooth image prior **α** as **β** approaches infinity. By virtue of (6), a reasonable value is *β* = 1, which was used for the work presented here.

### 2.5. Joint Estimation of Blood Input and Fatty Acid Metabolism

**Figure 4:**
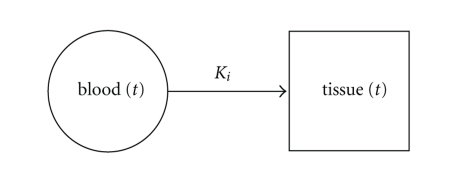
One-tissue-compartment model used for quantifying fatty acid metabolism during the first 90 s after injection of ^123^I-BMIPP.

To obtain a quantitative estimate of the metabolic rate of ^123^I-BMIPP in the myocardium, a one-tissue-compartment model (Figure 4) is fitted to TACs for higher-resolution voxels in a 7 × 7 × 7 neighborhood centered on the blood pool. Early myocardial tissue uptake is modeled with a single, irreversible compartment

(12) $$\text{tissue}\left(t\right)=K_{i}\cdot \overset{t}{\underset{0}{\int }}\text{blood}\left(\tau \right)d\tau ,$$

where *K*_*i*_ is the metabolic rate of ^123^I-BMIPP. Each voxel is modeled as a mixture of blood input and tissue uptake, taking into account partial volume effects

(13) $$\begin{array}{cc} \text{voxel}\left(t\right) & =\left[f_{v}\cdot \text{blood}\left(t\right)\right]\\ & \;+\left[\left(1-f_{v}\right)K_{i}\cdot \overset{t}{\underset{0}{\int }}\text{blood}\left(\tau \right)d\tau \right], \end{array}$$

where *f*_*v*_ is the fraction of vasculature in the tissue and also incorporates the effect of spillover from the blood pool to surrounding tissue voxels. For blood voxels, the factor (1 − *f*_*v*_)*K*_*i*_ incorporates the effect of spillover from surrounding tissue voxels.

Because of tissue spillover, there is no reconstructed voxel that contains a pure blood TAC; thus, the blood input function is assumed to be unknown and is modeled by a B-spline TAC

(14) $$B\left(t\right)=\overset{N}{\underset{n=1}{\sum }}b_{n}V^{n}\left(t\right).$$

The temporal B-spline coefficients {*b*_*n*_; *n* = 1,…, *N*} are jointly estimated with compartmental model parameters {(*f*_*v*_^*m*^, *k* ^*m*^); *m* ∈ *Ω*} for each voxel by minimizing the following least-squares criterion:

(15) $$\underset{m\in \Omega }{\sum }\overset{T}{\underset{0}{\int }}{\left\{{\hat{A}}^{m}(t)-\left[f_{v}^{m}B(t)+k^{m}\overset{t}{\underset{0}{\int }}B(\tau )d\tau \right]\right\}}^{2}dt,$$

where *Ω* denotes the 7 × 7 × 7 neighborhood of voxels centered on the blood pool, *T* = 90 s, and *k* ^*m*^ = (1 − *f*_*v*_^*m*^)*K*_*i*_^*m*^. Thus, there is a total of (2 · 7^3^) + 3 = 689 parameters to jointly estimate (i.e., two compartmental model parameters *f*_*v*_^*m*^ and *k* ^*m*^ for each of the 343 voxels in the neighborhood *Ω*, and three blood curve B-spline coefficients *b*_1_, *b*_2_, and *b*_3_).

The minimization proceeds by first initializing *B*(*t*) to the TAC for the voxel at the center of the neighborhood *Ω*. Then, optimal values for the B-spline coefficients {*b*_*n*_; *n* = 1,…, *N*} for *B*(*t*) are found with use of an iterative search algorithm. Note that one does not need to search explicitly for optimal values for the conditionally linear compartmental model parameters {(*f*_*v*_^*m*^, *k* ^*m*^); *m* ∈ *Ω*}, as there are unique optimal values for these parameters given *B*(*t*) [36, 37].

**Figure 5:**
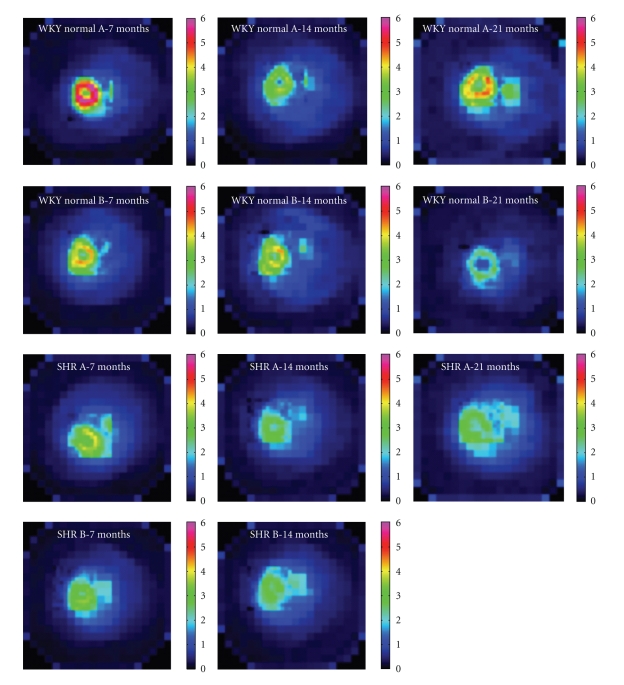
Typically, more trapping of ^123^I-BMIPP is evident in late 3D static images of the WKY normal hearts (top two rows), compared to the SHR hearts (bottom two rows). Trapping also tends to decrease with age (left column, 7 months; middle column, 14 months; right column, 21 months). SHR B died of congestive heart failure before 21 months. These static images have been normalized to one another by normalizing by the injected dose per unit body weight.

 Values for *K*_*i*_ reported in the results are based on the final estimate for *B*(*t*) and the average of TACs for 12 myocardial tissue voxels identified in static 3D images reconstructed from summed late data acquired 1.5–60 min after injection (Figure 5).

## 3. Results

In the late 3D static images (Figure 5), more trapping of ^123^I-BMIPP is evident in the WKY normal hearts (top two rows), compared to the SHR hearts (bottom two rows). These static images have been normalized to one another by normalizing by the injected dose per unit body weight.  Table 1 lists the body weight of each rat for each study.

**Figure 6:**
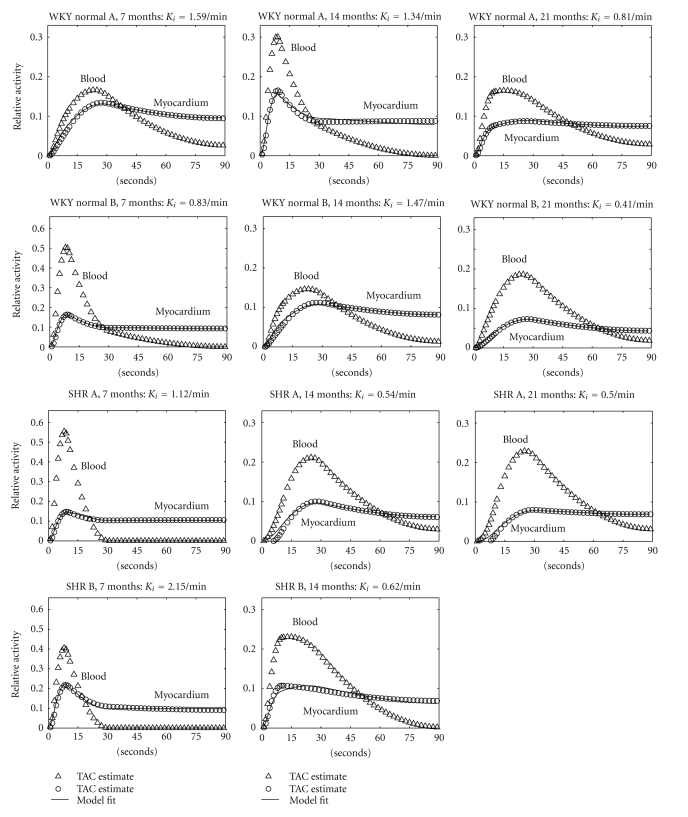
Time-activity curves for the WKY normal rats (top two rows) and the SHRs (bottom two rows) capture quantitative differences between their spillover-corrected blood inputs and myocardial uptakes (triangles and circles, resp.). Compartmental models (solid lines) provide good fits to the myocardial uptake curves. Left column, 7 months; middle column, 14 months; right column, 21 months. SHR B died of congestive heart failure before 21 months.

 For the early 4-D dynamic images, the use of nonuniform time sampling with splines that varied quadratically in time, along with the use of a smooth 4-D image prior, yielded smooth time-activity curves that captured the relatively fast rise and fall of ^123^I-BMIPP in the left ventricular blood pool, as well as the uptake and initial trapping of the radiotracer in the left ventricular myocardium.  Figure 6 shows time-activity curves for the spillover-corrected blood input function and myocardial uptake (triangles and circles, resp.), as well as the compartmental model fit (solid line) to the myocardial uptake curve, for each study.

**Figure 7:**
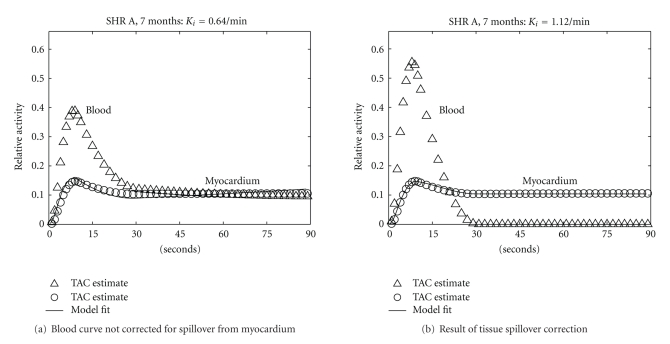
Time-activity curves for SHR A at 7 months estimated (a) without and (b) with tissue spillover correction for the blood curve. Spillover correction improves contrast between the blood input and myocardial uptake (triangles and circles, resp.), improves the fit of the compartmental model (solid line), and yields a metabolic rate estimate (*K*_*i*_) that nearly doubles, from 0.64 min^−1^ to 1.12  min^−1^.

The spillover of tissue activity into the left ventricular blood pool averaged 19 ± 10% across all 11 studies. Tissue spillover correction compensated for partial volume effects and improved the contrast between the blood input and myocardial uptake curves for all studies and visually improved the fit of the compartmental model for some studies (Figure 7). Metabolic rate estimates (*K*_*i*_) increased by an average of 72 ± 45% across all 11 studies, compared to estimates obtained without spillover correction.

**Figure 8:**
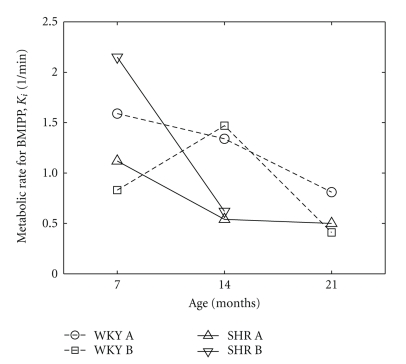
Metabolic rate of ^123^I-BMIPP in the myocardium as a function of age. SHR B died of congestive heart failure before 21 months.

 Estimates of *K*_*i*_ obtained from corrected blood curves are plotted as a function of age for all 11 studies in Figure 8. The general decline with age is what one expects. Slower rates of fatty acid metabolism in the SHRs at 14 months, compared to the WKY normal rats, is also expected as the SHR hearts switch to a reliance on glycolysis as the primary pathway for energy production during the development of heart failure. SHR B died of congestive heart failure before 21 months.

## 4. Discussion

We showed that it is potentially feasible to estimate the blood and myocardial tissue time-activity curves in rat models from projection measurements for dynamic data acquired with slow camera rotation of 1 s per projection, even when recirculation times are on the order of 6–8 s. The tissue spillover correction method compensated for partial volume effects and resulted in an increase of 72 ± 45% in estimates of metabolic rates *K*_*i*_, compared to estimates obtained without spillover correction. The results appear to indicate higher fatty metabolism in the control WKY rats as compared with SHRs. The fatty acid metabolic rate also decreases with age in both animal models.

The review of Nohara [1] describes how BMIPP behaves in normal and diseased hearts at the cellular and molecular level. BMIPP is a 15-carbon chain with a methyl group in the *β*-position which inhibits oxidative metabolism and reduces myocardial washout kinetics. In the cell, 60% of BMIPP will be retained mostly in the triglyceride (TG) pool and has a longer retention than triglyceride, and 10% will be more or less metabolized to be washed out of the cell. Other than metabolizing to TG, the metabolites of ^123^I-BMIPP are mostly intermediate or final metabolites in the mitochondria, where ^123^I-BMIPP is metabolized to p-^123^I-iodophenyl acetic acid (PIPA) by alpha-oxidation as the first step, followed by a beta-oxidation process to lactate. ^123^I-BMIPP reflects fatty acid uptake and the size of the lipid pool, and back diffusion of ^123^I-BMIPP and lactate production are good markers of ischemia. A 10-fold increase in the concentration of free fatty acid (FFA) in the blood will decrease ^123^I-BMIPP extraction by 25% and increase washout by 25%, whereas a 2-fold increase in the glucose concentration in the blood does not inhibit ^123^I-BMIPP uptake into the cell.

Lipid transport in the blood and in the cell is dependent upon it being bound to proteins or lipid proteins such as VLDL in the blood. Lipid is extracted into the cell by membrane proteins and is affected by albumin/FFA ratio in the blood. Higher lipid or FFA/albumin level results in greater uptake of lipid into the cell. Fatty acid binding protein (FABP) in the cytosole is one factor that regulates lipid flux. Lipid will be used either for oxidation, triglyceride or phospholipid formation. Carnitine palmityl transferese is the key enzyme for the entrance of lipid into mitochondria, and oxidative enzymes such as long chain acyl CoA dehydrogenase (LCAD) will determine lipid use into the TCA cycle. The eventual production of ATP in the mitochondria will limit the size of TG storage. The several lipid enzymes in the cell are regulated by nuclear genes that are individually activated by peroxisome proliferator activated receptors (PPARs). There are many endothelial proteins that are also involved in the storage and metabolism of lipid. The amount of medium chain acyl CoA dehydrogenase (MCAD) is closely related to the progression of heart failure. In the compensated phase of hypertrophy, m-RNA level of MCAD is reduced.

Even with species differences, our results (here for fatty acid metabolism, and [6, 7] for glucose metabolism) and other results obtained with the SHR model [38–40] corroborate studies in humans, which show that there are abnormalities in myocardial metabolic function manifested by reduced rates of fatty acid utilization and oxidation and an increase in glucose metabolism associated with hypertension-induced left ventricular hypertrophy (LVH) and idiopathic cardiomyopathy [21, 41]. In a separate positron emission tomography (PET) study imaging ^18^F-fluorodeoxyglucose (FDG) with use of a microPET scanner, we observed that glucose metabolism in the SHR model was greater than that in the WKY control [6, 7], and that the glucose metabolism in both decreased with age. Present results differ from results imaging fatty acid utilization in patients with heart failure [13], where it was shown that myocardial fatty acid uptake in patients with heart failure was higher than that expected for the normal heart, whereas myocardial glucose uptake rates were lower. It may be that in the hypertrophied heart before heart failure there is greater dependency on glucose and with eventual heart failure there is a shift to fatty acid metabolism, and that this shift is an indication of impaired energy efficiency in the failing heart.

Our work also corresponds to work by Okizaki et al. [5]. Using dynamic SPECT imaging of ^123^I-BMIPP, they showed that fatty acid metabolism was higher in patients with hypertrophic cardiomyopathy. Using mathematical modeling, they showed that compartmental model rate parameters might be a sensitive predictor for early detection of hypertrophic cardiomyopathy and could be a useful index to evaluate its progression. A two-compartment model was used where the first compartment was a reversible compartment (cytoplasma) and the second compartment (triglyceride pool) was an irreversible compartment corresponding to the long retention of BMIPP if incorporated into the triglyceride pool. The compartmental analysis suggested that a fatty acid shift (rate of transport into the TG pool higher than normal and a decreased volume of distribution for the first compartment) from cytoplasm to the triglyceride pool occurs in hypertrophic cardiomyopathy, even in the early phase of the disease when no apparent change was observed upon visual interpretation by nuclear medicine physicians. Because of the computational demands, we were able to model only the first 90 s of the kinetics in the myocardium. We used a single, irreversible tissue compartment, and therefore were able to measure only the uptake of ^123^I-BMIPP, which we consider to be a measure of the metabolic rate of ^123^I-BMIPP. It is anticipated that an additional, reversible tissue compartment will be needed to account for washout of ^123^I-BMIPP from the myocardium over longer time scales.

^123^I-BMIPP imaging changes in the ischemic condition [1]. Disparity between ^123^I-BMIPP uptake in the hypertrophied myocardium and delayed ^201^Tl redistribution images in rest/stress studies indicate that myocardial ischemia plays an important role in impaired fatty acid utilization and metabolism in hypertrophied myocardium [4]. Myocardial ischemia is present with hypertrophic cardiomyopathy for several reasons: small coronary artery disease, coronary artery spasms, left coronary artery compression, inadequate capillary density in relation to the increased myocardial mass, and impaired coronary flow reserve. Lipid metabolic regulation completely differs from normal in the very early phase of cardiac hypertrophy and with deteriorating heart failure, and metabolic switching from lipid to glucose will occur [1]. A slight reduction in flow will be reflected in an increase in glucose metabolism to a level 4-5 times the resting value, but the lipid metabolism will remain constant [1]. At less than 40% of resting flow, both lipid and glucose metabolisms decrease remarkably [1]. Extraction of lipids will decrease in cases of prolonged ischemia [1]. The myocardial accumulation of ^123^I-BMIPP is also related to ATP content, and thus may reflect pO2 levels [4]. Decreased myocardial ^123^I-BMIPP uptake in areas of stress-induced ischemia on ^201^Tl imaging indicated that exercised-induced metabolic changes persisted even in the resting condition [4]. The possibility of an unknown hereditary fatty acid metabolic abnormality that could account for reduced ^123^I-BMIPP accumulation in hypertrophic cardiomyopathy has also been reported [4].

Our studies show a decrease in fatty acid metabolism in both the SHR and WKY rat with age. In one study in humans it was observed that myocardial fatty acid utilization (MFAU) and myocardial fatty acid oxidation (MFAO) declined with age while myocardial glucose utilization (MGU) did not change; thus, the proportional contribution of glucose used to overall substrate utilization relative to fatty acids was increased [42]. The decline in MFAO and MFAU with age may reflect a decline in mitochondrial long-chain fatty acid uptake [43], and alterations in mitochondrial lipid content, composition, and protein interactions, leading to significant membrane dysfunction [44] and effects of impaired myocardial vasodilator capacity [45]. The study in humans [42] appears to correlate with some studies in mouse and rat experimental models that show that the contribution of MFAO to overall myocardial substrate metabolism declines with age, and that the proportion of glucose metabolism to overall substrate metabolism increases [46]. However, one study in rats [47] appears to contradict these results showing that during the transition from adulthood to senescence, there is an increase in palmitate (fatty acid) oxidation and a decrease in lactate oxidation, and that this is associated with significant deterioration in cardiac function and efficiency. This paper suggests that the metabolic changes occur in parallel with hypertrophy. Thus, if hypertrophy is involved there could be an accompanied disproportionate enlargement in cell length causing an increase in the cell length-to-width ratio [48]. Cell loss, which increases with age [49], may also play an increasing role in the transition to heart failure by placing a greater workload on the remaining viable cells. The detrimental effect of increased fatty acid utilization observed in the senescent heart may be attributed to the fact that fatty acids are a less efficient fuel in terms of myocardial oxygen consumption (MVO2) [50].

Limitations of our present study are (1) small sample size (two WKY, two SHR), (2) animals were not fasted, (3) no blood pressure data, (4) no independent validation of the input function either from blood samples or from simulation, (5) data were not corrected for attenuation or scatter, and (6) only the first 90 s of the dynamic data were processed. Regarding fasting, it was felt that the animals had fairly similar free fatty acid, insulin, and glucose levels, which we verified in another study in which we sampled blood. Blood pressure is very difficult to obtain in these animals. Presently, simulations with known blood input functions are underway to determine the bias and variance that one would obtain with the experimental data. In future studies we plan to implement attenuation and scatter correction using the transmission source on the GE Hawkeye SPECT/CT system. We have performed simulations that showed improvement in quantitation using scatter and attenuation correction [35]. In a rat it improves quantitation by 10–15%. Future work will also include addressing computational issues associated with reconstructing a 4-D dynamic image from the entire 60 min of projection data.

Our injected dose of 4 mCi (150 MBq) is high when compared with dose/weight ratios of what one would give a human. The injected dose was based on our past experience [8, 9] as to what we anticipated the photon counting statistics needed to be to perform the data analysis. Our statistical uncertainties appear to be reasonable. Nonetheless, this still needs to be evaluated in an extensive study of statistical precision based on injected dose in rats. We have not found any harmful effects to the animals using this dose; however, an extensive radiobiology study has not been performed.

The work presented here relates primarily to the development of technology for dynamic pinhole SPECT imaging of small animals. The results are the first to report on being able to measure fatty acid metabolic rate in rats with use of a clinical pinhole SPECT system with slowly-rotating detectors. From a technical standpoint, this measurement was made possible in part because of the slow (10–30 s) injection of ^123^I-BMIPP, which allowed good angular sampling of the projections of the blood input function. The slow injection was also necessary, so as not to overwhelm the rat's circulatory system with the additional volume (~1 mL) of the injectate. For faster injections of smaller volumes of injectate, an imaging system with stationary detectors, such as the U-SPECT II preclinical scanner [51], is better-suited for tomographic imaging of the resulting faster kinetics. The U-SPECT II also has the advantage of having a collimator with 75 1-mm pinholes for use with imaging rats, which provides about 13 times the sensitivity of our two-pinhole system and the ability to resolve 0.8-mm “cold” rods in a radioactive background [51]. Sensitivity and spatial resolution of our system could also be improved by judiciously increasing the number of and decreasing the size of the pinholes.

 Our methods can also be applied to other imaging modalities, such as dynamic PET. We are presently studying fatty acid metabolism as a function of age in SHR and WKY rats using microPET imaging of the fatty acid analog, 14(*R*, *S*)-[^18^F]fluoro-6-thia-heptadecanoic acid (^18^F-FTHA) [52]. Combining new dynamic and conventional clinical imaging protocols with improved descriptions of the heart (physiological, mechanical, and biochemical) will allow better specification of the heart's properties. This, in turn, will facilitate the study of how these properties are affected by molecular changes in the heart caused by disease.

##  Disclaimer

This document was prepared as an account of work sponsored by the United States Government. While this document is believed to contain correct information, neither the United States Government nor any agency thereof, nor The Regents of the University of California, nor any of their employees, makes any warranty, express or implied, or assumes any legal responsibility for the accuracy, completeness, or usefulness of any information, apparatus, product, or process disclosed, or represents that its use would not infringe privately owned rights. Reference herein to any specific commercial product, process, or service by its trade name, trademark, manufacturer, or otherwise, does not necessarily constitute or imply its endorsement, recommendation, or favoring by the United States Government or any agency thereof, or The Regents of the University of California. The views and opinions of authors expressed herein do not necessarily state or reflect those of the United States Government or any agency thereof or The Regents of the University of California.

## Figures and Tables

**Table 1:** Body weight of each rat for each study. SHR B died of congestive heart failure before 21 months.

	Body weight		
	7 Months	14 Months	21 Months
WKY normal A	420 g	482 g	545 g
WKY normal B	432 g	494 g	564 g
SHR A	430 g	465 g	416 g
SHR B	403 g	446 g	—
